# Independent contributions of family and neighbourhood indicators of socioeconomic status and migrant status to risk of mental health problems in 4–12 year old children

**DOI:** 10.1016/j.ssmph.2020.100675

**Published:** 2020-09-30

**Authors:** Mirte Boelens, Hein Raat, Junwen Yang-Huang, Gea M. Schouten, Amy van Grieken, Wilma Jansen

**Affiliations:** a Department of Public Health, Erasmus University Medical Center, PO Box 2040, 3000, CA, Rotterdam, the Netherlands; b Department of Research and Business Intelligence, Municipality of Rotterdam, P O Box 3001, Rotterdam, AH, the Netherlands; c Department of Social Development, Municipality of Rotterdam, PO Box 70032, LP, Rotterdam, the Netherlands

**Keywords:** Socioeconomic status, Children, Mental health problems, Neighbourhood, Migrant status, Material deprivation

## Abstract

**Rationale:**

A range of family and neighbourhood indicators of socioeconomic status and migrant status have been shown to be associated with risk of mental health l problems (MHP) in children. In this study we determined the independent contributions of these indicators.

**Objectives:**

The main objective is to examine independent associations of family and neighbourhood socioeconomic status indicators and migrant status with risk of MHP in children.

**Methods:**

We analyzed data from an anonymous public health survey among 5010 parents/caretakers of children aged 4–12 years living in Rotterdam, The Netherlands, gathered in 2018. Outcome of interest was risk of MHP measured using the total difficulties score of the Strengths and Difficulties Questionnaire. Associations of parent-reported perceived financial difficulties, material deprivation (not being able to provide certain goods, or leisure, educational or cultural activities or care use for children due to financial restrictions), parental educational level, child's migrant status and neighbourhood socioeconomic status with risk of MHP and with the total difficulties score were assessed using multilevel multivariable logistic and linear regression models.

**Results:**

In total, 473 (9.5%) children had a high risk of MHP. We observed independent associations of perceived financial difficulties, material deprivation and parental educational level with risk of MHP and with an increase in total difficulties score (P < 0.05). Migrant status and neighbourhood socioeconomic status were not independently associated with risk of MHP or a change in total difficulties score.

**Conclusions:**

Already in early life, perceived financial difficulties by parents, material deprivation reported by parents and lower parental education appeared to be independently associated with the risk of MHP in 4–12 year olds. Health professionals should be aware of the relatively higher risks in these subgroups and consider policies address this.

## Highlights

• Uses multilevel models to examine associations with child mental health problems.
• Mat erial deprivation increases risk of child mental health problems.
• Lower parental education increases risk of child mental health problems.
• Studies risk of SES indicators and migrant status on child mental health problems.

## Introduction

Approximately 10–20% of children and adolescents worldwide experience mental health problems (MHP) (Kieling et al., 2011). Onset of MHP usually occurs during childhood or adolescence with key MHP such as anxiety, depression, self-harm, ADHD (attention-deficit hyperactivity disorder), conduct disorders, PTSD (post-traumatic stress disorder) and eating disorders (Kessler et al., 2007; McGorry et al., 2011; Mental Health Foundation., 2019; Polanczyk et al., 2015). MHP in childhood may have a long lasting effect and track into adulthood (Kieling et al., 2011; Patel et al., 2007). Poor mental health is associated with multiple negative consequences such as employment difficulties, educational attainment and substance use later in life (Kieling et al., 2011; Patel et al., 2007). Timely awareness and intervention in childhood may reduce the severity and persistence of MHP (McGorry et al., 2011).

According to Cheng & Goodman (2015)and (Braveman et al, 2005) socioeconomic status is a multidimensional construct which consists of several different but highly related indicators such as income, education, employment and neighbourhood characteristics (Braveman et al., 2005; Cheng & Goodman, 2015). Migrant status is also highly related but not similar to socioeconomic status (Cheng & Goodman, 2015). Braveman et al., (2015) put forward that different socioeconomic status indicators affect health through distinct, possibly interacting, pathways at different levels (e.g. individual, family or neighbourhood) and therefore recommends studying different socioeconomic indicators (Braveman et al., 2005). Children from disadvantaged backgrounds or with a migrant status were found to have an increased risk of MHP (Minh et al., 2017; Pillas et al., 2014; Reiss et al., 2019; Sundquist et al., 2015).

Limited research has been performed to elucidate independent associations of socioeconomic indicators with MHP while this may increase our understanding of the differential, possibly interacting, pathways underlying these associations. These insights may give implications for policy making and practice.

Several cohort studies observed associations of financial difficulties, poverty or material deprivation (not being able to provide certain goods, or leisure, educational or cultural activities or care use for children due to financial restrictions) with MHP in children (Gunnarsdóttir et al., 2016; Rijlaarsdam et al., 2013; Wickham et al., 2017). For example a study performed in (N = 6330) children aged 4–16 from several Nordic countries demonstrated an association of financial difficulties with MHP in children (Gunnarsdóttir et al., 2016). A possible underlying mechanism is that perceived financial difficulties and material deprivation limit the goods and activities (for example leisure, educational or cultural activities or care use) that contribute to children's development, makes children different from their more affluent peers and in turn may influence mental health of children (Chaudry & Wimer, 2016). A second mechanism, the so-called family-stress model, suggests that perceived financial difficulties and material deprivation lead to parental stress affecting parental mental health and in turn their children's mental health (Chaudry & Wimer, 2016). Indeed, in the generation R cohort it was found that maternal depressive symptoms mediated the association of financial difficulties and material deprivation with mental health problems in their three-year-old children (N = 2169). Moreover, some parents with an income above the poverty line may still experience financial difficulties or material deprivation while other parents with a low income may not perceive financial difficulties (Huang et al., 2017). Therefore, perceived financial difficulties or material deprivation may be important indicators of socioeconomic status in the association with MHP in children.

Other studies have examined associations of parental educational level or migrant status with MHP in children (Arroyo-Borrell et al., 2017; de Laat et al., 2018; Geyer et al., 2006; Stevens & Vollebergh, 2008). Lower parental educational level or a non-Western migrant status were both associated with MHP in children (N = 3100) aged 4–14 years old in a Spanish cross-sectional study (Arroyo-Borrell et al., 2017). In a Dutch longitudinal study in children (N = 3410) aged 5–6 years old, lower-educated mothers reported more MHP in their children (de Laat et al., 2018). Parental educational level may be associated with differences in lifestyle, parenting choices, skills, and differences in providing adequate resources or help and in turn influence the MHP of their children (Geyer et al., 2006). Cultural differences due to migration, discrimination, differences in social position, asymmetrical acculturation within families and differences in parenting styles may directly or indirectly lead to a higher risk of MHP in children in migrant children (Stevens & Vollebergh, 2008).

Some studies have examined associations of neighbourhood indicators of socioeconomic status with MHP in children (Pillas et al., 2014). In a Swedish cohort study, an association of the socioeconomic status of the neighbourhood with a higher risk of MHP in children and adolescents was found (Sundquist et al., 2015). Whereas, in a British cohort study, in 10–15 year old children an association with risk of MHP was observed, but most variability was explained by family indicators of socioeconomic status (Jonsson et al., 2018). Previous literature has suggested that neighbourhoods with much poverty and/or unemployment may influence child MHP (Chaudry & Wimer, 2016; Curtis et al., 2013).

Earlier research has shown associations of a range of family and neighbourhood indicators of socioeconomic status and migrant status with risk of MHP in children. Yet it is unclear what their independent contribution is. In particular, insight in independent contributions of perceived financial difficulties and material deprivation n and their influence on children's risk of MHP is limited.

Therefore, the goal of this study was to examine independent associations of family (perceived financial difficulties, material deprivation, and parental educational level) and neighbourhood (neighbourhood socioeconomic status) indicators of socioeconomic status and of migrant status with risk of MHP in children. More insight in the independent contributions of these variables may have implications for better targeted preventive policies and practice aiming to reduce socioeconomic inequalities in youth MHP. In our study we will focus on 4–12 year old children as MHP can already be present at a young age and early intervention could reduce severity and persistence (Kessler et al., 2007; McGorry et al., 2011; Mental Health Foundation., 2019; Polanczyk et al., 2015).

## Research questions

1) Are family indicators or socioeconomic status (perceived financial difficulties, material deprivation and lower or intermediate parental educational level) independently associated with MHP in 4–12 year old children?
2) Is the socioeconomic status of the neighbourhood independently associated with MHP in 4–12 year old children?
3) Is a non-Western migrant status of the child independently associated with MHP in 4–12 year old children?
4) Is there an interaction effect of age, sex or family situation with indicators of socioeconomic status or with migrant status or between indicators or socioeconomic status and migrant status?

## Methods

### Study population and design

Data were used from a cross-sectional Dutch public health survey carried out in 2018 by the municipal public health service in the city of Rotterdam (Gezondheidsmonitor Kinderen GGD Rotterdam-Rijnmond). The survey targeted parents/caretakers of children aged 0–12 years old and questionnaires were filled out by the main caregiver. For our study we used survey data of parents of children aged 4–12 years old as the Strengths and Difficulties questionnaire (SDQ) to measure risk of MHP was not assessed in younger children. Invitations to participate were done by drawing a random probability sample from the municipal population register stratified by neighbourhood. Children who were living in a healthcare institution were excluded and parents received an invitation for one child only. All parents were living in Rotterdam when the survey was administered. Parents received hardcopy invitation letters with information about the survey and login details for the online survey. A hardcopy questionnaire was sent with the second time parents received the invitation. Parents could refuse to participate by not filling out the questionnaire. The survey data were collected by online or hardcopy questionnaires offered in Dutch, English and Turkish. Non-responders were contacted by telephone and were offered extra help in completing the questionnaire. Extra effort was made to target parents with Turkish and Moroccan backgrounds and residents of neighborhoods with a low response. Parents/caretakers of N = 5010 children aged 4–12 years old participated. The response rate was 34% and varied between 23 and 54% depending on the neighbourhood. Response did not differ by age or sex of the children. A comparison of parents of children with missing data (N = 1047; 20.9%) with parents of children with complete data (N = 3963; 79.1%) showed that parents of children with missing data less often reported material deprivation, were higher educated, more often from a Western migrant status or Dutch and more often lived in a neighbourhood with a lower socioeconomic status (P < 0.05). Parents of children with missing data did not differ according to perceived financial difficulties, family situation, sex, age, and total difficulties score (P > 0.05).

### Ethics

Our study relied on anonymous survey data collected in the context of performing statutory tasks (Public Health Act Netherlands). Observational research with anonymous data does not fall within the ambit of the Dutch Act on research involving human subjects and does not require the approval of an ethics review board. The Dutch Code of Conduct for Medical Research allows the use of anonymous data for research purposes without an explicit informed consent (Council of the Federation, 2018). Parents were free to refuse participation and could refuse by not filling out the questionnaire.

## Measures

### Perceived financial difficulties

Perceived financial difficulties was assessed by the question: Have you had difficulties in the past 12 months making ends meet with your household income? This question had four answer categories: no difficulty at all (60.6%), no difficulty but I do have to keep an eye on what I spend (24.1%), yes some difficulty (11.6%), and yes a lot of difficulty (3.7%). For the analyses the answers were dichotomized as either: no (no difficulty at all and no difficulty but I do have to keep an eye on what I spend) and yes (yes some difficulty and yes a lot of difficulty).

### Material deprivation

Eight statements assessed material deprivation (not being able to provide certain goods, or leisure, educational or cultural activities or care use for children due to financial restrictions):

1) my child cannot be a member of a sports club;
2) my child cannot be a member of another club such as theater or music;
3) my child cannot attend birthday parties or trips with school;
4) we cannot go on holiday or days-out;
5) my child cannot eat fruit or vegetables daily;
6) my child cannot attend swimming lessons;
7) my child cannot go to a care provider if that is actually necessary;
8) my child cannot receive the medication or care that is needed.

These statements are based on EU-SILC questions (Arora et al., 2015). The eight statements were transformed to a material deprivation score ranging from zero to eight, with eight being the highest score (i.e. parents could not afford any of the eight items). We did this by computing a score in which every statement answered with yes counted as a one and every statement answered with no as a zero. Internal consistency of the scale was good (Cronbach's alpha of 0.85). For the analyses the score was dichotomized to either no material deprivation or material deprivation (at least one of the eight items showed deprivation). We dichotomized the scale because the distribution of the scale was skewed (i.e. 0.5% of the parents could not afford any of the eight items (showed deprivation) to 77.9% of the parents who could afford all eight items).

### Migrant status

Migrant status was dichotomized as non-Western migrant (41.1%) or Western migrant together with Dutch natives (45.8%). Western migrants consisted of 13.1% of the total sample. We refer to this category as Western migrant and Dutch. A child was considered to have a non-Western migrant status as either the child was not born in a Western country or one or both parents were not born in a Western country. The following countries were considered Western: Europe (except Turkey), North America, Oceania, Indonesia and Japan in accordance with Statistics Netherlands (Statistics Netherlands, 2012).

### Parental educational level

Parental educational level was defined as highest educational level level obtained by one of the parents. Thus we categorized education based on the parent who obtained the highest education. Parental educational level was categorized as lower (no education, primary school or ≤ 4 years general secondary school), intermediate (>4 years general secondary school or intermediate vocational training) and higher education (higher vocational training, university degree or higher) (Statistics Netherlands, 2017).

### Neighbourhood socioeconomic status

The most recent data about the socioeconomic status of the neighbourhood (2017) were obtained from the Netherlands Institute of Social Research (SCP) (Netherlands Institute of Social Sciences, 2019). Matching to the questionnaire data was done using the neighbourhood code. The SCP computed a socioeconomic status score of the neighbourhood using principal component analysis based on mean adult income, percentage low adult incomes, percentage of low educated adult residents and percentage of unemployed adult residents in a neighbourhood. Previously, these scores have been found to be associated with health outcomes (de Boer et al., 2019; Vos et al., 2015). We computed tertiles of the neighbourhood socioeconomic status scores to create equal sized groups. The lowest tertile corresponds with a lower socioeconomic status score, the second tertile corresponds with an intermediate socioeconomic status score and the highest tertile corresponds to a higher socioeconomic status score. In total, 57 neighbourhoods in Rotterdam were included in our study. The amount of parents and children per neighbourhood in our sample varied from 30 to 160 with a mean of 88 (median = 84). Out of these 57 neighbourhoods we classified 18 as lower (32.7%; N = 1642), 18 as intermediate (30.7%; N = 1539), 13 as higher (21.%; N = 1054) socioeconomic status and 8 were not classified (15.5%; N = 775).

## Outcome assessment

### Risk of MHP

Risk of MHP in children was measured using the parent-strengths and difficulties questionnaire (SDQ) included in the Dutch Public health survey (Goodman, 2001). The SDQ is a validated tool (Goodman & Goodman, 2009; Theunissen et al., 2016). Internal consistency of the total difficulties score in our sample was adequate. (Cronbach's alpha of 0.73). The SDQ consists of the following domains: emotional problems, conduct problems, hyperactivity, peer problems, and prosocial behavior. A total difficulties score of the SDQ was calculated by adding the sub-scores of all domains except for the prosocial domain. The total difficulties score was dichotomized as either a high total difficulties score (above the cut-off for risk of MHP) or a not-at-risk score (below the cut-off) based on Dutch validated age-dependent cut-off values (Theunissen et al., 2016). For children aged 4–7 years old a total difficulties score of ≥15 indicates risk of MHP and for children aged 7–12 years old of the cut-off is ≥ 14 (Theunissen et al., 2016).

## Covariate assessment

Age, gender and family situation of the child were considered possible confounders based on literature and were derived from the survey (Reiss et al., 2019; Sundquist et al., 2015; Wickham et al., 2017). Age was measured in years. Gender was measured as a dichotomous variable using boy as reference category. Family situation was measured as two-parent family, single-parent family, or other situation using two-parent family as the reference.

## Statistical analyses

Characteristics are presented as means ± standard deviations (SDs) for continuous variables and as percentages for categorical variables for the total study population and stratified by risk of MHP (a high total difficulties score) and a not-at risk score. Differences in characteristics between children with a not-at-risk score or risk of MHP (a high total difficulties score) were computed using unpaired two-sample t-tests for continuous data and Chi-square tests for categorical data.

Missing data on covariates (ranging from 0.20% to 15.5%, see Table 1.) were imputed with SPSS using a fully conditional specified model based on the relationships between all the variables included in this study (M = 10 datasets). Pooled estimates were used to obtain the odds ratio's (OR), betas and corresponding 95% confidence intervals (CI) using the imputed data.

**Table 1:** Characteristics of the study population stratified by risk of MHP or not-at-risk total difficulties score in N = 5010 Children living in Rotterdam, the Netherlands in 2018.

		Total population	Not-at-risk score^a^	Risk of MHP score^a^	P-value
%, N		5010	90.5% 4509	9.5% 473	
Age		7.6 (2.3)	7.5 (2.3)	8.1 (2.1)	<**0.0001**
Gender	Boy	51.6 (2584)	50.4 (2271)	62.8 (297)	**<0.0001**
	Girl	48.4 (2426)	49.6 (2238)	37.2 (176)	
Family situation^b^	Two-parent	74.7 (3717)	75.8 (3395)	66.0 (310)	**<0.0001**
	Single-parent or other	25.3 (1256)	24.2 (1081)	34.0 (160)	
Perceived financial difficulties^c^	No	84.7 (4197)	86.1 (3846)	71.3 (330)	**<0.0001**
	Yes	15.3 (756)	13.9 (620)	28.7 (333)	
Material deprivation^d^	No	77.9 (3828)	79.5 (3525)	61.9 (283)	**<0.0001**
	Yes	22.1 (1086)	20.5 (910)	38.1 (174)	
Migrant status^e^	Western migrant and Dutch	58.9 (2938)	60.0 (2691)	49.4 (232)	**<0.0001**
	Non-Western migrant	41.1 (2046)	40.0 (1795)	50.6 (238)	
Parental educational level^f^	Higher	51.1 (2459)	52.9 (2299)	35.4 (155)	**<0.0001**
	Intermediate	32.3 (1554)	31.4 (1367)	40.9 (179)	
	Lower	16.6 (796)	15.7 (682)	23.7 (104)	
Neighbourhood socioeconomic status g	Higher	24.9 (1054)	25.6 (971)	19.7 (82)	**0.007**
	Intermediate	36.3 (1539)	36.5 (1385)	35.5 (148)	
	Lower	38.8 (1642)	38.0 (1441)	44.8 (187)	

Data expressed as M ± SD for continuous data and p-value computed using Unpaired T-tests. Data expressed as % for categorical data and p-value computed using chi-square tests. A high total difficulties score corresponds with a score above the age dependent cut off: score (e.g. for children aged 4–7 years old ≥15 and for children aged 7–12 ≥ 14).

MHP = mental health problems.

a 28 are missing (0.6%) .

b 37 are missing (0.7%).

c 57 are missing (1.1).

d 96 are missing (1.9%).

e 26 are missing (0.5%).

f 201 are missing (4.0%).

g 775 are missing (15.5).

To assess associations of perceived financial difficulties, material deprivation, parental educational level, migrant status and the socioeconomic status of the neighbourhood with risk of MHP (a high total difficulties score) in children we used multivariable multilevel logistic regression analyses. We used a random intercept and fixed slopes model to obtain the odds ratio's ORs and corresponding 95%CIs of having MHP (a high total difficulties score) as compared to a not-at-risk score. First we computed an intercept-only model to obtain the Median Odds Ratio (MOR). We also computed the MOR for the adjusted models. The MOR is used to examine the variance explained by the neighbourhood (Austin & Merlo, 2017). The MOR shows the median value of the distribution of the odds ratio between the neighbourhood with the lowest risk and the neighbourhood with the highest risk (Austin & Merlo, 2017; Larsen & Merlo, 2005). The MOR varies between 1 and infinity. If the MOR is 1 there is no variation between neighbourhoods (Larsen & Merlo, 2005). We adjusted for confounding using three models. Model 1 is a crude model and not adjusted for confounders. Model 2 is adjusted for age and gender of the child and for family situation. Model 3 is additionally adjusted for all other family level socioeconomic status indicators (perceived financial difficulties, material deprivation, educational level), migrant status and for the socioeconomic status of the neighbourhood to obtain the independent associations.

Subsequently, multivariable multilevel linear regression analyses were performed using the total difficulties score as continuous outcome measure. First we computed an intercept-only model to obtain the intraclass correlation (ICC). We also computed the ICC for our adjusted models. The ICC is used to examine the variability between neighbourhoods and ranges between 0 and 1. The closer the ICC is to 1 the more variability there is between neighbourhoods. We regarded an ICC smaller than 0.01 as negligible. We adjusted for confounding similarly as for the logistic multivariable multilevel regression analysis. All correlations stayed well below 0.7 and all variance inflation factor (VIF) values below 2. Two sided p-values denoted statistical significance (P < 0.05). IBM SPSS statistics for Windows, version 25.0 (International Business Machines Corporation, Armonk, New York) was used for all analyses.

## Results

Table 1 shows characteristics of the total sample and stratified by risk of MHP versus not-at-risk scores. A total of 473 (9.5%) children had risk of MHP (a high total difficulties score). Children with risk of MHP (a high total difficulties score) were more likely to be older, boy, to live in a single-parent or other family situation, to have a non-Western migrant status and to be from neighbourhoods with a lower socioeconomic status compared to children with a not-at-risk score (P < 0.05). These children were also more likely to have parents who perceived financial difficulties, had material deprivation or were intermediate educated (P < 0.05).

Table 2 presents results of the multilevel multivariable logistic regression analyses. The intercept-only model shows a MOR of 1.36 indicating some variation/heterogeneity between neighbourhoods in risk of MHP (high versus not-at-risk). Model 3 shows that parental perceived financial difficulties (OR 1.56; 95% CI: 1.18, 2.07), material deprivation (OR 1.53; 95% CI: 1.17, 1.99) and a lower (OR 1.74; 95% CI: 1.33, 2.28) and intermediate parental educational level (OR 1.69; 95% CI: 1.34, 2.13) were all significantly independently associated with risk of MHP (a high total difficulties score) compared to a not-at-risk score in children. Migrant status and the socioeconomic status of the neighbourhood were not significantly associated with risk of MHP (a high total difficulties score) in children after full adjustment in model 3.

**Table 2:** Multilevel associations of family and neighbourhood indicators of socioeconomic status and migrant status with risk of MHP in N = 5010 children living in Rotterdam, the Netherlands in 2018.

	**Null model**	**Model 1** OR (95%CI)	**Model 2** OR (5% CI)	**Model 3** OR (95% CI)
**Level 1: family**				
**Financial hardship**				
Yes		**2.39 (1.92, 2.96)**	**2.26 (1.80, 2.83)**	**1.56 (1.18, 2.07)**
No		Ref	Ref	Ref
**Material deprivation**				
Yes		**2.31 (1.89, 2.81)**	**2.18 (1.78, 2.69)**	**1.53 (1.17, 1.99)**
No		Ref	Ref	Ref
**Migrant status**				
Non-Western migrant		**1.50 (1.23, 1.82)**	**1.42 (1.17, 1.73)**	1.12 (0.91, 1.39)
Western migrant and Dutch		Ref	Ref	Ref
**Parental education**				
Lower		**2.29 (1.79, 2.94)**	**1.99 (1.55, 2.56)**	**1.74 (1.33, 2.28)**
Intermediate		**2.00 (1.61, 2.49)**	**1.88 (1.50, 2.34)**	**1.69 (1.34, 2.13)**
Higher		Ref	Ref	Ref
**Level 2: neighbourhood**				
**Neighbourhood socioeconomic status**				
Lower		1.28 (0.90, 1.82)	1.22 (0.88, 1.70)	1.02 (0.75, 1.39)
Intermediate		1.15 (0.81, 1.15)	1.10 (0.79, 1.53)	1.03 (0.75, 1.40)
Higher		Ref	Ref	Ref
Neighbourhood variance	0.10			0.05
**MOR**	1.36			1.25

OR = odds ratio and represent odds of MHP (a high total difficulties score) compared to a not-at-risk score (ref = not-at-risk score).

CI = confidence interval.

Numbers in **bold** are significant.

MOR = median odds ratio, sum; exp(sqrt(2*neighbourhood variance)*0.6745)).

^l^Model 1 is the crude, unadjusted model.

^2^Model 2 is adjusted for age, gender (ref = boy), and family situation (ref = two-parent family).

^3^Model 3 includes model 2 and additionally adjusted for perceived financial difficulties (ref = no), material deprivation (ref = no), parental educational level (ref = higher), migrant status (ref = Western migrant and Dutch) and for neighbourhood socioeconomic status (ref = higher).

Table 3 shows the results of the multilevel multivariable linear regression analyses. The ICC of the intercept-only model is 1.6% which is relatively low. Model 3 shows that all family level indicators of socioeconomic status were independently associated with the total difficulties score, namely: perceived financial difficulties (beta 1.50; 95% CI: 1.04, 1.97) material deprivation (beta 1.37; 95% CI: 0.94, 1.79) and a lower (beta 0.98; 95% CI: 0.45, 1.51) and intermediate educational level (beta 0.68; 95% CI: 0.27, 1.08). Migrant status and neighbourhood socioeconomic status were not associated with a higher total difficulties score after full adjustment in model 3 (P > 0.5).

**Table 3:** Multilevel associations family and neighbourhood indicators of socioeconomic status and migrant status with the total difficulties score in N = 5010 children living in Rotterdam, the Netherlands in 2018.

	Null model	Model 1 Change in total difficulties score (95% CI)	Model 2 Change in total difficulties score (95% CI)	Model 3 Chance in total difficulties score (95% CI)
**Level 1: family**				
**Perceived financial difficulties**				
Yes		**2.71 (2.33, 3.08)**	**2.54 (2.16, 2.92)**	**1.50 (1.04, 1.97)**
No		Ref	Ref	Ref
**Material deprivation**				
Yes		**2.46 (2.13, 2.78)**	**2.30 (1.96, 2.64)**	**1.37 (0.94, 1.79)**
No		Ref	Ref	Ref
**Migrant status**				
Non-Western migrant		**0.63 (0.33, 0.92)**	**0.54 (0.24, 0.83)**	−0.00 (−0.33, 0.33)
Western migrant and Dutch		Ref	Ref	Ref
**Parental education**				
Lower		**1.62 (1.15, 2.10)**	**1.37 (0.88, 1.87)**	**0.98 (0.45, 1.51)**
Intermediate		**1.14 (0.77, 1.52)**	**1.01 (0.63, 1.38)**	**0.68 (0.27, 1.08)**
Higher		Ref	Ref	Ref
**Level 2: neighbourhood**				
**Neighbourhood socioeconomic status**				
Lower		0.73 (−2.46, 3.20)	0.30 (−2.48, 3.08)	0.15 (−1.55, 1.85)
Intermediate		0.20 (−1.22, 1.62)	0.18 (−1.03, 1.39)	0.03 (−1.02, 1.08)
Higher		Ref	Ref	Ref
Random effect variance	0.39			0.07
Residual effect variance	23.86			22.75
**ICC (ICC%)**	0.016 (1.6%)			0.003 (0.3%)

CI = confidence interval.

Numbers in **bold** are significant.

ICC = intraclass correlation, sum; random effect variance/(random effect variance + residual effect variance). Here the percentage is given which is the ICC*100%.

Change in total difficulties score is the difference (beta) in total difficulties score on the SDQ.

^l^Model 1 is the crude, unadjusted model.

^2^Model 2 is adjusted for age, gender (ref = boy) (ref = bref), and family situation (ref = two-parent family).

^3^Model 3 includes model 2 and additionally adjusted for perceived financial difficulties (ref = no), material deprivation (ref = no), parental educational level (ref = higher), for migrant status (ref = Western migrant and Dutch) and for neighbourhood socioeconomic status (ref = higher).

Sensitivity analyses using a complete case sample of N = 3963 participants yielded similar results for the risk of a high total difficulties score and for change in total difficulties score (See Table SA and SB). However, besides perceived financial difficulties, material deprivation, and parental education, also a lower socioeconomic status of the neighbourhood is significantly associated with a change in the total difficulties score (beta 0.52; 95% CI: 0.04, 0.99). Interaction effects were explored between all socioeconomic status indicators and migrant status. Interaction effects were also explored between age and gender of the child, family situation and socioeconomic status indicators and migrant status. After applying the Bonferroni correction for multiple testing (P = 0.05/25 = 0.002), no significant interaction was found.

## Discussion

In this large population based cross-sectional study in 4–12 year old children, we found that children with parents who perceived financial difficulties, reported material deprivation or who obtained lower or intermediate education had a higher risk of MHP.

The prevalence of children with risk of MHP (a high total difficulties score) in our sample is in agreement with the prevalence of around 10–20% of children of this age experiencing MHP worldwide (World Health Organization, 2019).

Perceived financial difficulties and material deprivation are both independently associated with risk of MHP in children in our study. Our findings are supported by other studies performed in the United Kingdom, United States of Americaand Sweden (Kirby, Wright, & Allgar, 2019; Kim & Hagquist, 2018; Neckerman et al., 2016). A possible explanation may be that not only poverty or quantitative income but also the experience of financial difficulties and material deprivation play a role (Alegría et al., 2018). Although perceiving financial difficulties or experiencing material deprivation may be more common among parents living below the poverty line, some of these parents may be able to make the ends meet, for example because they have lower expenses or because they get support from others or by community organizations (Huang et al., 2017). Thus those parents may not perceive financial difficulties or material deprivation. Furthermore, some families living above the poverty line have for example higher expenses and less support and therefore may perceive financial difficulties or experience material deprivation. Perceiving financial difficulties or experiencing material deprivation may lead to stress and impact parental mental health and in turn increase the risk of MHP in their child (Chaudry & Wimer, 2016). Indeed, in our sample 18.0% of the parents who perceive financial difficulties do not experience material deprivation and 11.7% of the parents who experience material deprivation do not perceive financial difficulties.

We also found an association of lower and intermediate parental education with risk of MHP in children. Associations of parental educational level with risk of MHP were also found in earlier research (Boe et al., 2014; de Laat et al., 2018; Hosokawa & Katsura, 2018). Parental educational level was found to influence parental mental health through stress (Reiss, 2013). Lower parental educational level was also found to influence, parenting skills and choices such as lax, hostile or over reactive parenting (Boe et al., 2014; de Laat et al., 2018; Parkes et al., 2015; Suzuki et al., 2019). Parental mental health, parental stress and parenting practices may influence the mental health of children (de Laat et al., 2018). Yet, the exact pathway is still unclear.

We found no independent association of migrant status with risk of MHP. Earlier research found mixed results regarding migrant status (Stevens & Vollebergh, 2008). One possible explanation for these mixed results is that the effect of migration varies between certain migrant groups (Stevens & Vollebergh, 2008). If so, studying different migrant groups as on group may lead to masked results. Another possible explanation is that differences in MHP in children with different migration backgrounds are due to socioeconomic circumstances (Stevens & Vollebergh, 2008). Indeed, after we adjusted our model for family socioeconomic indicators the association of migrant status with risk of MHP was no longer significant. At last, there may be cultural differences in what is perceived as MHP (Stevens & Vollebergh, 2008).

We found no association of a lower or intermediate neighbourhood socioeconomic status with risk of MHP. Other studies found associations of the socioeconomic status of the neighbourhood with a higher risk of MHP (Enticott et al., 2016; Reijneveld et al., 2010; Sundquist et al., 2015). In these studies, the age of the study population was on average older. It might be that for younger children familial circumstances and not neighbourhood circumstances are more important for mental health than for adolescents or adults. Another possible explanation is that younger children have less exposure to the neighbourhood, whereas older children or adolescents are able to experience the neighbourhood more and are more exposed to neighbourhood deprivation affecting their mental health (Boardman & Saint Onge, 2005).

Strengths of our study include the large sample size, a validated questionnaire to assess risk of MHP, the population-based setting and most importantly, a range of different socioeconomic status indicators. A key limitation is that due to the cross-sectional design of this study, no causation or temporal direction of the associations can be established. The survey data were not nationally representative and therefore the generalizability of our findings may be reduced. Moreover, our survey had a response rate of 34% which could lead to selection bias in our sample. Interestingly, (Davern et al, 2010) suggest that higher response rates do not automatically result in different estimates (Davern et al., 2010). We did not measure other family or neighbourhood indicators of socioeconomic status such as income or work situation. Using other indicators of socioeconomic status or different categorizations of indicators may lead to somewhat different results. The measure of the socioeconomic status of the neighbourhood has been used in previous research and was found to be associated with perinatal morbidity and health care costs (de Boer et al., 2019; Vos et al., 2015). However, no information regarding the validity or reliability of this measure is present. We based our study on survey data about topics that may be seen as sensitive such as MHP, perceived financial difficulties and material deprivation thus we need to be cautious of social desirability bias influencing the estimates. Finally, even though we adjusted for confounders, residual confounding might be present because of possible incompletely or unmeasured confounders.

This study contributes to the evidence that different indicators of socioeconomic status are independently associated with risk of MHP in children. Children growing up with parents who perceive financial difficulties, report material deprivation and have lower educational levels have a higher risk of MHP. We recommend more research, preferably using longitudinal designs, to replicate our findings. For the development and implementation of effective preventive interventions and policies it is important to unravel possible distinct pathways for socioeconomic inequalities for MHP in children. Future studies applying mediation analyses could provide further insight in these pathways.

We observed distinct independent associations of family indicators of socioeconomic status with risk of MHP in children as young as 4–12 years old. Health professionals should be aware of the relatively higher risks in children with parents who perceive financial difficulties, material deprivation or who are lower or intermediate educated. Preventive interventions and policies should adequately address the specific needs of these particular subgroups and realize sufficient reach among them using insights on prevention of MHP in children (World Health Organization, 2013). Preventive policies to reduce material deprivation of families, for example by in-kind-support policies, might be a promising way to improve mental health in children (Huang et al., 2017).

## Conclusion

We observed independent associations of perceived financial difficulties, material deprivation and parental educational level with an increased risk of MHP in children. Further research is warranted to confirm our findings and to unravel possible pathways underlying these associations with risk of MHP in children. To prevent MHP in children, policies and measures that target parents with lower or intermediate education or aimed to reduce parental perceived financial difficulties and material deprivation may be of importance.

## Data availability

Data used were obtained from a Dutch Public Health survey carried out in 2018 by the municipal public health service in the city of Rotterdam (Gezondheidsmonitor Kinderen GGD Rotterdam-Rijnmond). The data used in this study are protected by the Municipal Health Service of Rotterdam. Data are available under request via: gezondheidsmonitorbco@rotterdam.nl. Data on neighbourhood socioeconomic status were obtained from the Netherlands Institute for Social Research.

## CRediT authorship contribution statement

**Mirte Boelens:** Conceptualization, Methodology, Formal analysis, Writing - original draft. **Hein Raat:** Supervision, Conceptualization, Methodology, Writing - review & editing. **Junwen Yang-Huang:** Conceptualization, Writing - review & editing. **Gea M. Schouten:** Investigation, Writing - review & editing. **Amy van Grieken:** Conceptualization, Writing - review & editing. **Wilma Jansen:** Supervision, Conceptualization, Methodology, Writing - review & editing.

## Abbreviations

MHP: Mental Health Problems
ADHD: Attention Deficit Hyperactivity Disorder
PTSD: Post Traumatic Stress Disorder
SDQ: Strengths and Difficulties Questionnaire
SD: Standard Deviation
CI: Confidence Interval
OR: Odds Ratio
MOR: Median Odds Ratio
ICC: Intraclass Correlation
VIF: Variance Inflation Factor
