# Supplementary material for: Independent contributions of family and neighbourhood indicators of socioeconomic status and migrant status to risk of mental health problems in 4–12 year old children
Source: SSM Popul Health. 2020 Sep 30;12:100675. doi: 10.1016/j.ssmph.2020.100675 (PMC7548441; doi:10.1016/j.ssmph.2020.100675)
Supplement: Multimedia component 1 [file mmc1.docx]

**Supplemental tables**

| **Table SA.** Multilevel associations of socioeconomic indicators and migrant status with risk of MHP in N=3,963 4-12 year olds. | | | | |
| --- | --- | --- | --- | --- |
|  | **Null model** | **Model 1**  OR (95%CI) | **Model 2** OR (5% CI) | **Model 3**  OR (95% CI) |
| **Level 1: family** |  |  |  |  |
| **Perceived financial difficulties** |  |  |  |  |
| Yes |  | **2.39 (1.87, 3.06)** | **2.31 (1.79, 2.98)** | **1.51 (1.10, 2.08)** |
| No |  | ref | ref | ref |
| **Material deprivation** |  |  |  |  |
| Yes |  | **2.44 (1.95, 3.06)** | **2.36 (1.87, 2.98)** | **1.69 (1.26, 2.27)** |
| No |  | ref | ref | ref |
| **Migrant status** |  |  |  |  |
| Non-Western migrant |  | **1.46 (1.17, 1.83)** | **1.40 (1.12, 1.75)** | 1.08 (0.85, 1.38) |
| Western migrant and Dutch |  | ref | ref | Ref |
| **Parental education** |  |  |  |  |
| Lower |  | **2.11 (1.58, 2.83)** | **1.90 (1.41, 2.57)** | **1.52 (1.11, 2.08)** |
| Intermediate |  | **1.92 (1.50, 2.47)** | **1.85 (1.43, 2.39)** | **1.55 (1.18, 2.02)** |
| Higher |  | ref | ref | ref |
| **Cultural background** |  |  |  |  |
| **Level 2: neighbourhood** |  |  |  |  |
| **Neighbourhood socioeconomic status** |  |  |  |  |
| Lower |  | **1.72 (1.19, 1.48)** | **1.63 (1.13, 2.33)** | 1.23 (0.86, 1.76) |
| Intermediate |  | 1.32 (0.91, 1.93) | 1.26 (0.87, 1.82) | 1.08 (0.76, 1.55) |
| Higher |  | ref | ref | ref |
| Neighbourhood variance | 0.10 |  |  | 0.05 |
| **MOR** | 1.35 |  |  | 1.24 |
| ^1^ Model 1 is the crude, unadjusted model.  ^2^ Model 2 is adjusted for age, gender (boy = ref), and family situation (re f= two-parent family).  ^3^ Model 3 includes model 2 and additionally adjusted for perceived financial difficulties (ref = no), material deprivation (ref = no), parental educational level (ref = higher), migrant status (ref = Western migrant and Dutch) and for neighbourhood socioeconomic status (ref = higher). OR=odds ratio and represent odds of a high total difficulties (e.g. MHP) score compared to a not-at-risk score (ref= not-at-risk score). CI=confidence interval.  Numbers in **bold** are significant.  MOR=median odds ratio, sum; exp(sqrt(2*neighbourhood variance)*0.6745)). | | | | |

| **Table SB.** Multilevel associations of socioeconomic indicators and migrant status with the total difficulties score on the SDQ in N=3,963 4-12 year olds. | | | | |
| --- | --- | --- | --- | --- |
|  | **Null model** | **Model 1**  Change in total difficulties score (95%CI) | **Model 2** Change in total difficulties score (5% CI) | **Model 3**  Chance in total difficulties score (95% CI) |
| **Level 1** |  |  |  |  |
| **Financial hardship** |  |  |  |  |
| Yes |  | **2.59 (2.18, 3.01)** | **2.43 (2.01, 2.85)** | **1.35 (0.84, 1.86)** |
| No |  | ref | ref | ref |
| **Material deprivation** |  |  |  |  |
| Yes |  | **2.46 (2.11, 2.82)** | **2.31 (1.95, 2.68)** | **1.48 (1.03, 1.93)** |
| No |  | ref | ref | ref |
| **Migrant status** |  |  |  |  |
| Non-Western migrant |  | **0.54 (0.22, 0.87)** | **0.47 (0.15, 0.79)** | -0.09 (-0.41, 0.24) |
| Western migrant and Dutch |  | ref | ref | Ref |
| **Parental education** |  |  |  |  |
| Lower |  | **1.48 (1.04, 1.90)** | **1.24 (0.81, 1.68)** | **0.76 (0.31, 1.20)** |
| Intermediate |  | **1.14 (0.79, 1.49)** | **1.00 (0.64, 1.35)** | **0.61 (0.26, 0.97)** |
| Higher |  | Ref | ref | Ref |
| **Level 2** |  |  |  |  |
| **Neighbourhood socioeconomic status** |  |  |  |  |
| Lower |  | **1.13 (0.60, 1.66)** | **0.99 (0.48, 1.51)** | **0.52 (0.04, 0.99)** |
| Intermediate |  | 0.43 (-0.10, 0.97) | 0.35 (-0.17, 0.87) | 0.10 (-0.35, 0.56) |
| Higher |  | ref | ref | ref |
| Random effect variance | 0.39 |  |  | 0.078 |
| Residual effect variance | 23.86 |  |  | 22.38 |
| **ICC (ICC%)** | 0.016 (1.6%) |  |  | 0.003 (0.3%) |
| Change in total difficulties score is the difference (beta) in total difficulties score on the SDQ. ^1^ Model 1 is the crude, unadjusted model.  ^2^ Model 2 is adjusted for age, gender (ref = boy), and family situation (ref = two-parent family).  ^3^ Model 3 includes model 2 and additionally adjusted for perceived financial difficulties (ref = no), material deprivation (ref = no), parental educational level (ref = higher), for migrant status(ref = Western migrant and Dutch) and for neighbourhood socioeconomic status (ref = higher). CI=confidence interval.  Numbers in **bold** are significant.  ICC=intraclass correlation, sum; random effect variance / (random effect variance+ residual effect variance). Here the percentage is given which is the ICC*100%. | | | | |
